# Supplementary material for: Optochemical control over mRNA translation by photocaged phosphorodiamidate morpholino oligonucleotides in vivo
Source: Nat Commun. 2025 Apr 16;16:3614. doi: 10.1038/s41467-025-58207-5 (PMC12003629; doi:10.1038/s41467-025-58207-5)
Supplement: Supplementary file 2 — Reporting Summary [file 41467_2025_58207_MOESM2_ESM.pdf]

Corresponding author(s): Erez Raz
Surajit Sinha

Last updated by author(s): Feb 24, 2025

Reporting Summary

Nature Portfolio wishes to improve the reproducibility of the work that we publish. This form provides structure for consistency and transparency in reporting. For further information on Nature Portfolio policies, see our Editorial Policies and the Editorial Policy Checklist.

Statistics

For all statistical analyses, confirm that the following items are present in the figure legend, table legend, main text, or Methods section.

n/a	Confirmed	
☐	☒	The exact sample size (*n*) for each experimental group/condition, given as a discrete number and unit of measurement
☐	☒	A statement on whether measurements were taken from distinct samples or whether the same sample was measured repeatedly
☐	☒	The statistical test(s) used AND whether they are one- or two-sided<br>*Only common tests should be described solely by name; describe more complex techniques in the Methods section.*
☐	☒	A description of all covariates tested
☐	☒	A description of any assumptions or corrections, such as tests of normality and adjustment for multiple comparisons
☐	☒	A full description of the statistical parameters including central tendency (e.g. means) or other basic estimates (e.g. regression coefficient) AND variation (e.g. standard deviation) or associated estimates of uncertainty (e.g. confidence intervals)
☐	☒	For null hypothesis testing, the test statistic (e.g. *F*, *t*, *r*) with confidence intervals, effect sizes, degrees of freedom and *P* value noted<br>*Give P values as exact values whenever suitable.*
☒	☐	For Bayesian analysis, information on the choice of priors and Markov chain Monte Carlo settings
☒	☐	For hierarchical and complex designs, identification of the appropriate level for tests and full reporting of outcomes
☒	☐	Estimates of effect sizes (e.g. Cohen's *d*, Pearson's *r*), indicating how they were calculated

Our web collection on statistics for biologists contains articles on many of the points above.

Software and code

Policy information about availability of computer code

Data collection	Non applicable
Data analysis	ImageJ, Prism, Excel software packages were used

For manuscripts utilizing custom algorithms or software that are central to the research but not yet described in published literature, software must be made available to editors and reviewers. We strongly encourage code deposition in a community repository (e.g. GitHub). See the Nature Portfolio guidelines for submitting code & software for further information.

Data

Policy information about availability of data

All manuscripts must include a data availability statement. This statement should provide the following information, where applicable:

- Accession codes, unique identifiers, or web links for publicly available datasets
- A description of any restrictions on data availability
- For clinical datasets or third party data, please ensure that the statement adheres to our policy

Data availability statement is provided

Research involving human participants, their data, or biological material

Policy information about studies with human participants or human data. See also policy information about sex, gender (identity/presentation), and sexual orientation and race, ethnicity and racism.

Reporting on sex and gender	n/a
Reporting on race, ethnicity, or other socially relevant groupings	n/a
Population characteristics	n/a
Recruitment	n/a
Ethics oversight	n/a

Note that full information on the approval of the study protocol must also be provided in the manuscript.

Field-specific reporting

Please select the one below that is the best fit for your research. If you are not sure, read the appropriate sections before making your selection.

- [x] Life sciences
- [ ] Behavioural & social sciences
- [ ] Ecological, evolutionary & environmental sciences

For a reference copy of the document with all sections, see nature.com/documents/nr-reporting-summary-flat.pdf

Life sciences study design

All studies must disclose on these points even when the disclosure is negative.

Sample size	Sample size was determine based on the throughput of the experiment which was limited by the speed of embryo development
Data exclusions	no data exclusion was done
Replication	at least three repeats were conducted for each experiment
Randomization	n/a
Blinding	The same clutch of embryos was randomly divided among the experimental groups

Reporting for specific materials, systems and methods

We require information from authors about some types of materials, experimental systems and methods used in many studies. Here, indicate whether each material, system or method listed is relevant to your study. If you are not sure if a list item applies to your research, read the appropriate section before selecting a response.

Materials & experimental systems

n/a	Involved in the study	
[ ]	[x]	Antibodies
[ ]	[x]	Eukaryotic cell lines
[x]	[ ]	Palaeontology and archaeology
[ ]	[x]	Animals and other organisms
[x]	[ ]	Clinical data
[x]	[ ]	Dual use research of concern
[x]	[ ]	Plants

Methods

n/a	Involved in the study	
[x]	[ ]	ChIP-seq
[ ]	[x]	Flow cytometry
[x]	[ ]	MRI-based neuroimaging

Antibodies

Antibodies used	Purified Rabbit Anti- Activated Caspase-3, Clone C92-605 (RUO), BD biosciences, Cat#559565
Validation	published in https://doi.org/10.1016/j.devcel.2022.07.008

Eukaryotic cell lines

Policy information about cell lines and Sex and Gender in Research

Cell line source(s)	standard laboratory HeLa cell line was used, purchased from NCCS Pune repository
Authentication	N/A
Mycoplasma contamination	Cell lines were not tested for mycoplasma
Commonly misidentified lines (See ICLAC register)	N/A

Animals and other research organisms

Policy information about studies involving animals; ARRIVE guidelines recommended for reporting animal research, and Sex and Gender in Research

Laboratory animals	Zebrafish, Danio rerio
Wild animals	N/A
Reporting on sex	N/A
Field-collected samples	N/A
Ethics oversight	The research and animal handling are controlled by the local authorities of the city of Muenster and the state of NRW

Note that full information on the approval of the study protocol must also be provided in the manuscript.

Plants

Seed stocks	N/A
Novel plant genotypes	N/A
Authentication	N/A

Flow Cytometry

Plots

Confirm that:

- [x] The axis labels state the marker and fluorochrome used (e.g. CD4-FITC).
- [x] The axis scales are clearly visible. Include numbers along axes only for bottom left plot of group (a 'group' is an analysis of identical markers).
- [ ] All plots are contour plots with outliers or pseudocolor plots.
- [ ] A numerical value for number of cells or percentage (with statistics) is provided.

Methodology

Sample preparation	Cells were grown in 12-well plates to 50% confluency for 24h (37°C, 5% CO_2), dissociated (0.25% Trypsin-EDTA) and sorted.
Instrument	BD FACS Aria III
Software	BD FACSDiva, FlowJo software, and Graph Pad Prism 6.
Cell population abundance	All the cells are of the same line and relevance. The procedure was used only for the purpose of measuring signal leve, not actual sortingl.
Gating strategy	The first gating was performed using FSC-A versus FSC-W to exclude cells with unusual or smaller sizes, including potential debris. For the second gating, SSC-A versus SSC-W was applied to select cells with moderate complexity. The third gating

involved FSC-A versus SSC-A, with cells on the extreme edges being excluded. The gated population from FSC-A versus SSC-A was then visualized in a histogram, where the X-axis represents fluorescence intensity and the Y-axis shows the cell count. The unstained control sample was first processed using the same gating strategy, and a boundary was set at the right end of the histogram peak to distinguish between positive and negative staining cell populations. Consequently, the population shifting towards the right of this boundary represented the positively stained cells, while the population to the left represented the negatively stained cells.

☒ Tick this box to confirm that a figure exemplifying the gating strategy is provided in the Supplementary Information.
